# Predictors of Readmission after the First Acute Coronary Syndrome and the Risk of Recurrent Cardiovascular Events—Seven Years of Patient Follow-Up

**DOI:** 10.3390/life13040950

**Published:** 2023-04-04

**Authors:** Cristiana Bustea, Delia Mirela Tit, Alexa Florina Bungau, Simona Gabriela Bungau, Vlad Alin Pantea, Elena Emilia Babes, Larisa Renata Pantea-Roșan

**Affiliations:** 1 Department of Preclinical Disciplines, Faculty of Medicine and Pharmacy, University of Oradea, 410073 Oradea, Romania; 2 Department of Pharmacy, Faculty of Medicine and Pharmacy, University of Oradea, 410028 Oradea, Romania; 3 Doctoral School of Biomedical Sciences, University of Oradea, 410087 Oradea, Romania; 4 Department of Dental Medicine, Faculty of Medicine and Pharmacy, University of Oradea, 410073 Oradea, Romania; 5 Department of Medical Disciplines, Faculty of Medicine and Pharmacy, University of Oradea, 410073 Oradea, Romania

**Keywords:** recurrent cardiovascular events, predictors, hospital readmission, first acute coronary syndrome, complete revascularization, STEMI

## Abstract

Recurrent hospitalization after acute coronary syndromes (ACS) is common. Identifying risk factors associated with subsequent cardiovascular events and hospitalization is essential for the management of these patients. Our research consisted in observing the outcomes of subjects after they suffered an acute coronary event and identifying the factors that can predict rehospitalization in the first 12 months and the recurrence of another acute coronary episode. Data from 362 patients admitted with ACS during 2013 were studied. Recurrent hospitalizations were retrospectively reviewed from medical charts and electronic hospital archives over a period of seven years. The mean age of the studied population was 64.57 ± 11.79 years, 64.36% of them being males. The diagnosis of ACS without ST elevation was registered in 53.87% of the patients at index hospitalization. More than half had recurrent hospitalization in the first year after the first ACS episode. Patients with lower ejection fraction (39.20 ± 6.85 vs. 42.24 ± 6.26, *p* < 0.001), acute pulmonary edema during the first hospitalization (6.47% vs. 1.24%, *p* = 0.022), coexistent valvular heart disease (69.15% vs. 55.90%, *p* = 0.017), and three-vessel disease (18.90% vs. 7.45%, *p* = 0.002) were more frequently readmitted in the following twelve months after their first acute coronary event, while those with complete revascularization were less frequently admitted (24.87% vs. 34.78%, *p* = 0.005). In multiple regression, complete revascularization during the index event (HR = 0.58, 95% CI 0.35–0.95, *p* = 0.03) and a higher LVEF (left ventricular ejection fraction) (HR = 0.95, 95% CI 0.92–0.988, *p* = 0.009) remained independent predictors of fewer early readmissions. Complete revascularization of the coronary lesions at the time of the first event and a preserved LVEF were found to be the predictors of reduced hospitalizations in the first year after an acute coronary event.

## 1. Introduction

Cardiovascular diseases (CVD) represent the main factor in increasing incidence of death among the population, becoming the most common cause of death worldwide [1]. Ischemic coronary artery diseases (CADs) are among the cardiovascular diseases with increased risk of death, through the appearance of acute coronary syndromes (ACS). Acute myocardial ischemia, which results from inadequate blood flow across the coronary tree, determines the cluster of symptoms known as ACS [2]. Acute thoracic discomfort, which can be characterized as pressure, pain, burning, or tightness, is the primary sign that starts the diagnostic and therapeutic process in patients with presumed ACS. Dyspnea, discomfort in the epigastric area, and left arm pain are typical signs that are comparable to pain in the chest [3].

Three distinct categories of medical symptoms are typically covered by ACS. These are classified based on the ST-segment on the electrocardiogram (ECG) trace as non-ST-segment elevation, such as unstable angina (UA) and non-ST-segment elevation myocardial infarction (NSTEMI), or as ST-segment elevation, such as acute ST-segment elevation myocardial infarction (STEMI) [4].

ACS occurs several years after the development and progression of atheromatous plaque [5]. Atherosclerosis is inextricably linked to development/appearance of vulnerable atherosclerotic plaques and of ACS.

After formation, the atheromatous plaques may be stable for a long time. Their destabilization due to the appearance of a rupture of a vulnerable atheromatous plaque leads to ACS. The rupture of the atheromatous plaque induces platelet activation that results in the formation of the initial white thrombus, and then of the red thrombus, due to the hematologic elements in the fibrin network, which can partially or completely block the coronary artery lumen. Significant partial blockage of the lumen causes UA or NSTEMI, while complete blockage causes STEMI [6].

An unhealthy lifestyle, associated with non-modifiable CVD risk factors (like gender, age, genetic factors) or modifiable ones (like dyslipidemia, hypertension, diabetes, obesity, chronic kidney disease (CKD), or psycho-emotional stress), facilitates and stimulates atheromatous plaques [7]. Unhealthy diet/eating habits, a sedentary lifestyle, and/or smoking are associated with high plasma total cholesterol levels which trigger the atherogenic cascade and, in time, the development of CAD with the appearance of ACS [8]. Furthermore, the findings of a recent investigation revealed that a body mass index (BMI) of more than 25 kg/m^2^ is a significant indicator for increased on-treatment platelet reactivity in patients with STEMI receiving dual antiplatelet medication with ticagrelor or prasugrel and is correlated with tardy pharmacodynamic response to oral third-generation P2Y12 inhibitor loading dose [9].

Despite recent improvements in ACS treatment, CAD remains a major public health problem. Patients having ACS can be considered as also having a much higher risk (both short- and long-term) of recurrent CV events and hospitalizations. The possibility of such a recurrent CV event occurrence, or even the death of the subject, is most likely immediately after occurrence of ACS or in the next 12 months [10,11,12], and continues to be elevated in the following few years [12,13]. Comparing the data of a representative US population with an ACS event as the follow-up criterion, the event rate/5 years (representing non-fatal stroke or MI, or cardiovascular death) was 33.4%. Immediately following the index ACS, the risk was ≈six-fold higher compared to more than a year after leaving the hospital [14].

The overall goal of the management of patients with ACS is to re-establish and stabilize coronary blood flow and to initiate appropriate treatment to reduce the likelihood of recurrent CV events [15]. To prevent readmission and recurrent ACS, evidence-based post-discharge recommendations including medication, patient education, cardiac rehabilitation, and regular follow-up are required. Evidence from representative ACS populations from a current clinical practice setting will help in identifying strategies for improving patient outcomes. Considering the above-mentioned points, it is mandatory to understand in detail not only the studied populations’ characteristics, the chances of risk with the passage of time, the perception of the patterns, and the results of the current medical practice, but also the method for choosing the secondary prevention strategy (which is optimal for additional risk mitigation).

Therefore, we decided to focus on observing the short-/long-term evolution of subjects who had suffered an acute coronary event and identifying those factors that can predict rehospitalization in the first twelve months after ACS due to a recurrence of another acute coronary episode. In order to give importance to this research and to provide relevant results, the observations took place over seven years. This topic is rarely discussed in the literature, especially with this approach. The study design allows the identification of factors associated with readmission as well as the determination of independent predictors of reduced hospitalizations in the first year after an acute coronary event. The obtained findings can assist healthcare professionals in deciding what steps to take to lessen the possibility of readmission for patients who are affected.

## 2. Materials and Methods

### 2.1. Patient Selection

This retrospective study was carried out by following 362 patients who had been admitted to the Oradea County Emergency Clinical Hospital, Unit for Advanced Monitoring and Treatment of Critical Cardiac Patients for ACS, between 1 January 2013 and 31 December 2013. The outcome and recurrent hospitalizations were reviewed from medical charts and the electronic hospital archive during a 7-year period, until 30 June 2020.

The study included patients who presented with ACS in 2013 and who were readmitted at least once in the following 7 years. The exclusion criteria used for patient selection were as follows:

- Patients who had stable angina pectoris;
- Patients with ACS at the hospital admission moment who could not be dynamically monitored due to insufficient data;
- Patients with oncological conditions (they are at risk of cardiovascular complications due to cancer treatment that can influence the rate of hospital readmission [16]), or psychiatric conditions (which imply a reduced compliance with treatment that can influence recurrent hospitalization rate [17]) at the time of hospitalization for ACS;
- Pregnant or lactating patients.

**Figure 1:**
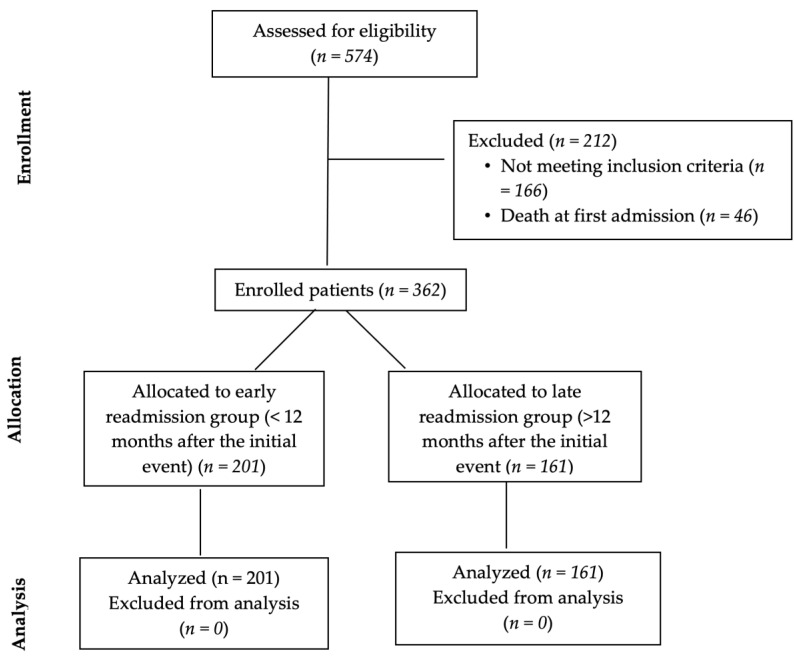
Type CONSORT diagram.

Initially, 574 patients were considered for potential inclusion in the study. However, only 362 patients met both of the aforementioned inclusion criteria. Figure 1 contains the flow chart describing the patient selection process. All 362 patients were readmitted at least once during the 7 years of follow-up. They were analyzed by comparing early (1 year) vs. late (more than 1 year) rehospitalizations.

ACS was diagnosed referencing the current European Society of Cardiology (ESC) Clinical Practice Guidelines. Acute myocardial infarction (AMI) was diagnosed when the following criteria were met:

- Typical angina clinical picture included suffering from myocardial ischemia accompanied by a rise in biomarkers of myocardial necrosis above the 99th percentile of the upper reference;
- Considering the level of the electrocardiographic (ECK) trace: new major left bundle branch block, ST-segment elevation, or the presence of a new Q wave;
- Checking the ECG, the presence of parietal kinetic disorders was observed;
- Angiographically: total occlusion of the coronary artery affected in the infarction [18].

Differentiation of STEMI from NSTEMI is based on ECG trace criteria. Thus, STEMI is considered to be present when a new ST-segment elevation or J-point elevation ≥1 mm is observed on the ECG trace in at least two contiguous leads other than V2 and V3, with the following particularities regarding V2–V3 leads:

▪ ≥2 mm (in men >40-years-old);
▪ ≥2.5 mm (in men <40-years-old);
▪ ≥1.5 mm (in women).

NSTEMI is considered to have occurred when ST-segment elevation ≥ 0.5 mm in two contiguous leads or when negative T-wave > 1 mm in two contiguous leads is present on the ECG trace [18]. At the same time, recurrent myocardial infarction is considered to be an AMI that occurs more than 28 days after a first AMI [18]. Unstable angina is considered to be part of ACS with no ST-segment elevation, along with NSTEMI; the difference between the two is based on the presence of myocyte necrosis revealed by the elevated levels of myocardial necrosis enzymes in the case of NSTEMI [19].

All patients were discharged after the first hospitalization with medication recommended by ESC guidelines for secondary prevention after acute coronary syndrome consisting of statins, P2Y12 inhibitors, β-blockers, aspirin, and angiotensin-receptor blockers or angiotensin-converting enzyme inhibitors.

### 2.2. Statistical Analysis

Excel (2019 version) tabulated data were processed using the SPSS statistical package (version 25 statistical software) [20]. The obtained results are described as mean ± SD (for continuous variables), and as frequencies and **%** (for categorical variables). Additionally, the 2 groups were compared through the independent sample *t*-test (in the case of continuous variables) or the Kruskal–Wallis test (in the case of the categorical variables). The Pearson bivariate correlation test allowed the analysis of the relationship between the continuous variables, and the Spearman bivariate correlation test was performed for the categorical variables.

In the case of significantly different parameters for the 2 groups with rehospitalization in the first year and more than one year after the first event, a multiple-regression type analysis was used in order to calculate each parameter’s value as an independent predictor for readmission in the first 12 months (*p* < 0.05 being considered statistically significant).

## 3. Results

A total of 362 patients was enrolled in the study; 233 were males; the mean age was 64.57 ± 11.79 years at first hospitalization for ACS. Regarding the type of ACS at first presentation, the diagnosis of ACS without ST elevation was more frequent, found in 195 patients (53.87%). For the group of patients with ACS without ST elevation, most had unstable angina (UA).

At the first hospitalization for ACS, patients presented with various cardiovascular risk factors and/or history of cardiovascular diseases. Most of them had dyslipidemia and hypertension, and many of them had diabetes and CKD (Table 1). A history of previous (old) myocardial infarction was registered in 21.5% of patients, and a large proportion of them had valvular heart disease and heart failure, or history of stroke (Table 1).

Coronarography was performed in 56% of patients and revealed mostly single-vessel disease. The artery most frequently affected was the left anterior descending artery, corresponding to the involvement of the anterior wall of the left ventricle and with ECG changes in the anterior territory. Most of the patients were treated with interventional revascularization, and only a limited number of patients with STEMI were subjected to thrombolysis. The patients with thrombolytic reperfusion therapy refused interventional investigation and treatment (10.5%). Five patients originating from geographic areas without hospitals containing percutaneous intervention (PCI) facilities were initially treated with thrombolytic therapy and then transferred to our hospital, where interventional revascularization was completed (they were eventually included in the PCI group).

During the first episode of hospitalization, the most frequent complications were rhythm disturbances, followed by cardiogenic shock and acute pulmonary edema (Table 2). A reduced number of patients presented at first admission with a hemorrhagic complication possibly related to antiplatelet, anticoagulant thrombolytic therapy: six patients had UGB, and 0.98% had a hemorrhagic stroke (Table 2).

**Figure 2:**
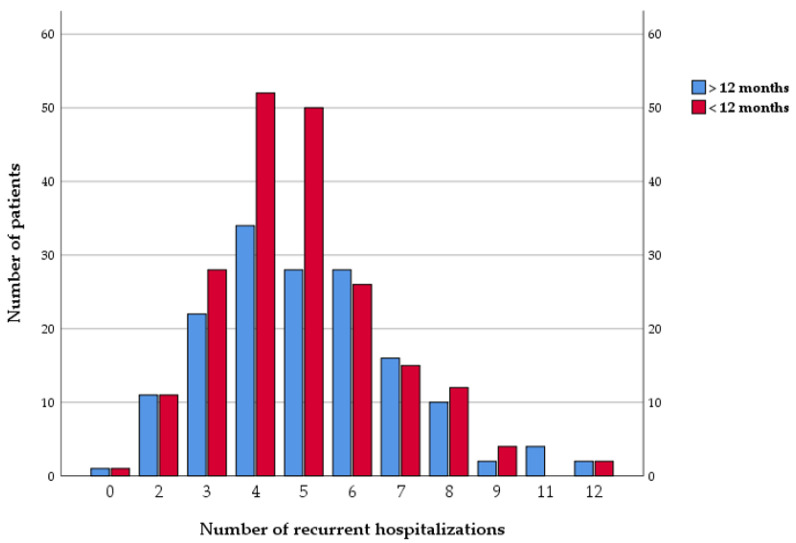
Number of recurrent hospitalizations.

During a follow-up period of seven years, all the patients were readmitted at least once. They registered a mean number of 4.46 ± 2.37 rehospitalizations during follow-up. More than half of the patients had recurrent hospitalization in the first year after the first ACS episode, and 44.5% of the patients were readmitted more than 12 months after the first acute coronary event (Figure 2).

No statistically significant differences existed between patients readmitted in the first 12 months compared to those readmitted after the first year, regarding associated cardiovascular risk factors. Patients with acute heart failure (specifically, acute pulmonary edema) during the first hospitalization were more frequently readmitted in the following 12 months after the first acute coronary event. Moreover, those with early readmission in the first 12 months had lower ejection fractions and more frequently associated valvular diseases (Table 3).

Angiographic characteristics at first admission that significantly correlated with recurrent hospitalization in the first 12 months were three-vessel involvement and incomplete revascularization during the first interventional procedure. 

**Figure 3:**
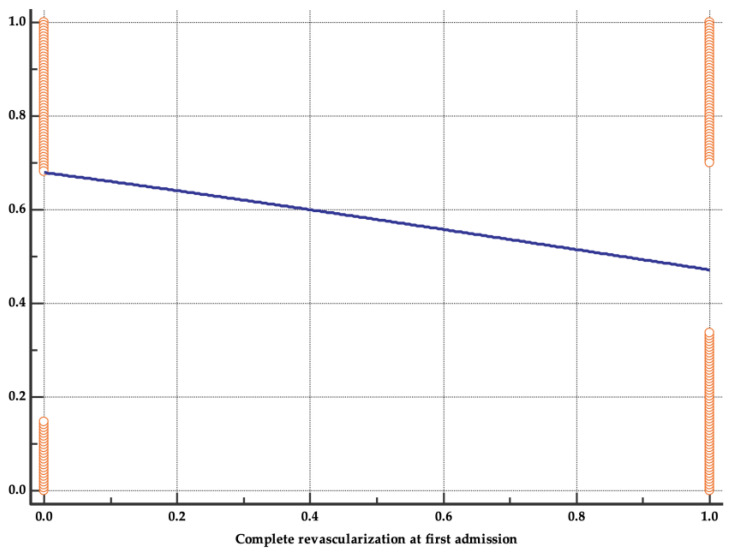
Regression analysis for complete revascularization procedure.

Multiple regression was used to analyze parameters significantly different between the two groups. Independent predictors for fewer early readmissions in the first 12 months after a first episode of ACS remained complete revascularization procedure at first hospitalization (HR = 0.58, 95% CI 0.35–0.95, *p* = 0.03) and a higher LVEF (HR = 0.95, 95% CI 0.92–0.988, *p* = 0.009) (Figure 3).

In 151 patients (41.71%), the second hospitalization readmission was due to ACS. The type of ACS was STEMI in 26 (17.21%) patients, NSTEMI in 22 patients (14.57%), and UA in 103 patients (68.21%). Of those patients with second admission due to STEMI, 12 patients (46.15%) presented in the first year after the first acute coronary event. Of the patients with NSTEMI, 12 (54.54%) were readmitted in the first year after the first acute coronary event. For most of the patients who presented with UA at the second admission (69 (66.99%)), symptoms occurred in the first 12 months after the first event.

Readmission for STEMI diagnosis was significantly correlated with both the existence of three-vessel CAD and the diagnosis of UA at first admission. Moreover, patients readmitted for STEMI had a lower ejection fraction at initial evaluation (Table 4). Readmission with NSTEMI diagnosis was more common in those having a previous old MI in their medical history, with two-vessel CAD, or with revascularization limited to the culprit lesion during first admission to the hospital.

Readmission for UA was more common in those subjects with associated heart failure, a lower ejection fraction, and associated CKD (Table 4). UGB at first hospitalization was significantly associated with readmission for STEMI or NSTEMI, probably due to inadequate dual antiplatelet therapy in the context of hemorrhagic complications during the first hospitalization. The obtained results indicate a weak-to-average correlation between the studied variables, which is nevertheless significant from a statistical point of view. Thus, it can be stated that the association between the variables is found at the same intensity, both in the sample and in the entire population.

## 4. Discussion

Because of its prevalence and the consequent elevated risk of repeated ischemic cardiovascular events, ACS remains a significant difficulty for specialists. Patients still experience a surprisingly increased risk of early repetitive ischemic episodes after ACS, regardless of recent improvements in both medical and interventional treatments.

There were fewer women than men with ACS in the present study, but no significant difference resulted between the two sexes with respect to recurrent hospitalization in the first 12 months after the index event. Although CHD is traditionally considered a male disease, clinical data (including reinfarction rates and MI mortality) reveal worse outcomes in women [21]. Similar results were presented in another observational multicenter retrospective study that included 1308 women and 2437 men. Both in the short and long term, no significant differences were found between the sexes, neither related to mortality, nor regarding the combined end point (reinfarction, cardiogenic shock, bleeding, stroke, or death) [22]. Additionally, a group of subjects from Canada, hospitalized with ACS and under follow-up for up to 2 years, highlighted the increased risk of adverse clinical outcomes in the case of women with ACS (who benefited from an early, invasive strategy, and from coronary revascularization) vs. men, although these differences were not seen in those treated with medical therapy alone [23].

Almost half of the ACS patients were initially admitted with STEMI, followed by UA, and only after that by NSTEMI. However, if ACS were categorized based only on the presence/absence of ST-segment elevation, the ratio of patients reverses, with more than half of them presenting with ACS without ST-segment elevation. Most studies on ACS define ACS as with/without ST-segment elevation and thus consider the frequency of non-ST-segment elevation ACS to be predominant, when, in fact, the differentiation of UA from NSTEMI in the case of ACS without ST-segment elevation is mostly omitted. This ignores the importance of the incidence of STEMI, though the presence of patients with STEMI is much higher compared to patients who develop NSTEMI or UA [24,25,26].

According to McManus DD [27], the incidence rates of STEMI are continuously declining compared to the previous data, while the incidence of NSTEMI has started to increase significantly lately. Neumann JT [28] believes that both the incidence rates of STEMI and UAP are declining, and only the incidence rates of NSTEMI are increasing.

However, the incidence rates of STEMI continue to be high among patients with CAD, but one should not ignore the incidence of ACS without ST-segment elevation which, as our research also shows, presents an increased ratio in terms of the incidence of patients with NSTEMI and UAP.

According to Wang TKM [29], NSTEMI patient mortality rates are low, despite the increased incidence of non-ST-elevation ACS vs. STEMI or UA patient mortality rates, which are increasing. 

Percutaneous or surgical coronary intervention for myocardial revascularization was indicated for patients with STEMI and NSTEMI, respectively, and less indicated for patients with UAP. Thus, most patients underwent PCI for myocardial revascularization, and only a very few patients underwent CAGB. According to Spadaccio [30], the decision to perform PCI vs. CAGB must be made by the heart team, which assesses the individualized risks and benefits for the patient [31]. However, there was no significant difference in terms of in-hospital mortality and survival among patients who underwent PCI or CAGB [32].

Thrombolysis was performed in less than a quarter of the patients, because our medical center owns a catheterization laboratory where PCI can be carried out and, as current guidelines [33] recommend, thrombolysis was carried out only in patients transferred from other medical centers where coronary angiography could not be performed and in patients who refused interventional therapy. Short- and long-term outcomes proved to be more favorable in subjects who underwent PCI, compared to cases where thrombolytic therapy was performed [34].

The anterior topographic territory was the preferred territory of myocardial infarctions (regardless of ST-segment elevation), followed by the inferior territory. Having the LAD (left anterior descending artery) as the epicardial coronary artery responsible for acute myocardial infarction is consistent with existing data that also implicate the LAD, along with the anterior territory, as responsible for the majority of MI [35,36,37].

Most patients who underwent PCI presented with single-vessel CAD, followed by two-vessel CAD, where the left coronary artery and the LAD-circumflex artery, respectively, were mainly involved. Less than a quarter of the patients presented with three-vessel ischemic coronary disease. These subjects were recommended to undergo CAGB if they presented with UAP upon hospital admission and revascularization of the culprit lesion in case they presented with MI. They were recommended to undergo subsequent complete myocardial revascularization by surgical revascularization of the remaining lesions.

All patients included in the study presented with classic cardiovascular risk factors or were known to have a personal history of cardiovascular pathology.

Dyslipidemia was the most frequent cardiovascular risk factor involved in the incidence of ACS and, at the same time, in the development of atheromatous plaques, being present in three quarters of the patients. Arterial hypertension is another important cardiovascular risk factor involved in atherogenesis, being present in more than half of the patients. Published data [38] are even more emphatic than that, stating that dyslipidemia and arterial hypertension are predictors of ACS, especially of the occurrence of UAP, followed by ACS + ST-segment elevation (specifically of STEMI rather than of NSTEMI).

Both diabetes mellitus and CKD are not at all negligible in the development of ischemic coronary diseases, as their involvement in the steps leading to the onset of ACS is known [39,40]. Existence of CAD in subjects who continued to present with ACS is another predictor of the development of this pathology [41]. Thus, the study also included patients with previous MI and previous stroke. Ischemic cardiovascular disease occurred quite frequently among patients who subsequently suffered a case of ACS, and mortality among these patients remained high [5,41,42].

Most patients admitted for ACS presented with congestive heart failure and significant valve regurgitations. Heart failure is considered to be frequently encountered together with ischemic coronary disease; gradually, through the presence of advanced atherosclerosis, the endothelial dysfunction present in patients with an increased risk of ACS will aggravate the already existing myocyte injury [43]. ACS is a precipitant of acute heart failure. These patients require special care, because, in the absence of prompt treatment, the mortality rate in this category of patients is high [44].

In ACS patients, the accurate determination of LVEF is extremely important, as impaired LVEF represents a predictor of an unfavorable short-term outcome, showing increased mortality 1 year after the first ACS event [45].

Arrhythmias occur frequently in patients with ACS, especially in those who develop an MI. The emergency requires prompt treatment, as it is known that these patients are more prone to malignant arrhythmias which, in the absence of adequate treatment, can lead to death [46,47].

The rate of readmission within a year was higher compared to readmission after more than one year after an acute coronary event. An increased risk of a recurrent cardiovascular event was also observed in numerous other published studies, especially in the first year after a case of ACS [10,11,12]. The risk of a nonfatal MI, nonfatal ischemic stroke, or cardiovascular death at 5 years after a first ACS was 33.4%, being six-fold higher in the first year after ACS compared with more than 1 year after the index ACS event in a very large US study [14]. Published research [10] shares the findings with our research, stating that the risk of ACS recurrence after a first acute episode is high.

ACS recurrence is also common within a year after onset in the OACIS (Osaka Acute Coronary Insufficiency Study) registry. The results of the study emphasized that the incidence of recurrent MI/year decreased from the first year (2.65%) in all subsequent years, up to 5 years (0.91–1.42%) [48]. Namiuchi et al. revealed in their research that the recurrence rate of ACS in the second year after a first MI (2.1%) was lower than in the year immediately following the MI (4.2%), concluding that the high recurrence rate can persist in cases having multiple MIs [49].

The Optum database (recording 239,234 patients with evidence of an ACS hospitalization over 14 years, beginning in 2005) revealed these patients as having a hugely increased risk (6.4% after the first year), in the short-term, after ACS hospitalization. This observation provides an additional argument for the utility of the guideline-based treatments strategies to be initiated during hospitalization for ACS [14].

The results of a study conducted in 2016 concluded that approximately 25% of patients who survive a case of ACS will suffer a stroke, AMI, or cardiovascular death in the next 5 years, particularly in the first year (34.8% risk) [10].

In the first year following discharge, 18.3% of the patients experienced an AMI, stroke, or cardiovascular death, according to a Swedish study (>90,000 patients with AMI), of whom around 50% had undergone revascularization. Of patients who had no occurrences during the first year of follow-up, 20% experienced an event after three years [50]. In the same direction, 21,890 individuals with a background of ACS were included in the REACH registry between 2003 and 2004, being monitored over the next five years (until 2009). Outcomes at one and four years showed that the overall incidence of stroke, AMI, or cardiovascular mortality was roughly 6% and 16%, respectively [51]. Between 2005 and 2010, another study on >15,000 United Kingdom patients resulted in a cumulative incidence of stroke, AMI, and cardiovascular death of 7.3% (at one year), 12.3% (after the second year) and 17.7% at three years [52]. All the studies above imply that there is a high probability of serious cardiovascular problems recurring following a case of ACS.

There were significantly more patients who experienced a rehospitalization in less than 12 months after the first one if they experienced three-vessel disease and incomplete revascularization during the index event, compared to patients who developed the event after 1 year from the first event. This may have happened because the culprit lesion was treated in the first ACS event, and the remaining lesions were to be revascularized later. However, due to other events, the remaining lesions were deferred, and, subsequently, patients developed a second ACS event within a year after the first ACS event. Rathod KS [53] claims that performing complete coronary revascularization at a safe stage is far superior to revascularizing only the culprit lesion, as patients who undergo complete revascularization have a better long-term prognosis compared to patients in whom the other stenotic lesions were deferred or revascularized later.

Another extensive analysis that should be mentioned, carried out between 2004 and 2005, refers to the so-called Melbourne Interventional Group registry. There are 9615 patients who received PCI for the index MI. In the next year after their index PCI, 12.2% of the patient surviving to discharge had a history of ACS or unplanned revascularization requiring hospitalization. Following them for 10 years, it was found that the number of unplanned hospitalizations decreased significantly, the rate of hospitalization/12 months falling from 15.3 to 7.6% (*p* < 0.001). Additionally, for hospitalization, the authors detailed in the study several independent predictors both in cases with recurrent ACS and in cases with unplanned revascularization, as follows: female sex, multivessel CAD, LV dysfunction, heart failure, diabetes, sleep apnea, etc. Finally, the paper concluded that, in the case of multi-vessel CAD subjects, optimizing therapeutic management of LV dysfunction, non-culprit vessel PCI, or diabetes can prevent hospitalization [54], these disorders being identified as predictors for recurrent admission within 12 months in our study as well.

Patients with severe heart failure (acute pulmonary edema during first hospitalization), associated valvular diseases, and lower ejection fraction were more frequently hospitalized during the first month after ACS.

Thus, the short-term prognosis for patients with ACS is unfavorable, especially in the first 12 months after the ACS event. According to the specialized literature, more than half of the patients with a first ACS event require hospitalization within the first year, and most of them require lifetime readmission due to an acute cardiovascular event [55,56,57,58].

Two-vessel or three-vessel disease, reduced LVEF after myocardial revascularization, acute or chronic heart failure, or upper gastrointestinal bleeding present in patients hospitalized with ACS are predictors of hospital readmission due to a new acute cardiovascular event.

**Figure 4:**
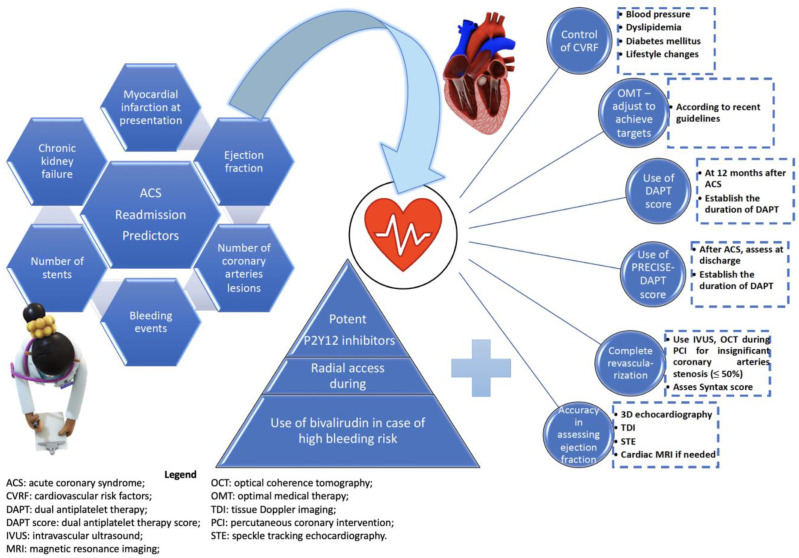
Main parameters and predictors of readmission in acute coronary syndrome subjects and a proposed algorithm for improving the outcome.

Figure 4 suggests a strategy for the management of the main predictors associated with second readmission in patients with ACS, according to the recommendations developed by the most current guidelines. The objective of these strategies is to optimize the evolution of the subjects, and the interventions are intended to influence the main predictors or complications that are associated with a negative prognosis.

By highlighting factors associated with readmission, as well as independent predictors of reduced hospitalizations in the first year after an acute coronary event, the results obtained in this study provide relevant information for clinicians. These data also allow the optimization of both the clinical management of patients with ACS and their evolution, through interventions that can influence the main predictors or complications that are associated with negative prognosis.

### Limitations and Strengths

Our study has some limitations that require consideration. The limited number of patients from a single center and the retrospective design together represent a significant shortcoming. The absence of specific data regarding the patients’ adherence to their medication regimens as well as potential follow-up failure are additional drawbacks. Only the city’s emergency public hospital underwent the readmission evaluation; neither other hospitals in the city nor hospitals in other regions were considered. These variables might have caused the number of readmissions to be underestimated. The benefit of this solely hospital-based strategy, on the other hand, is that it favored prospective enrollment of all eligible patients who had their first hospitalization for ACS and their close monitorization for a such a long period of seven years.

## 5. Conclusions

ACS represents the main cause of mortality and morbidity among patients with cardiovascular diseases. The incidence of STEMI increases in patients with ACS, but the incidence of non-ST-segment elevation acute coronary syndrome predominates both in patients developing ACS for the first time and in patients experiencing a second acute coronary event. Short- and long-term results in ACS patients remain unfavorable, most patients requiring rehospitalization within less than 12 months. Complete revascularization of the coronary lesions from the first event and a preserved LVEF were found to be the independent predictors of reduced hospitalizations in the following year after an acute coronary event.

## Figures and Tables

**Table 1:** Baseline first-admission characteristics of patients enrolled in the study.

Demographic Baseline Characteristics	No. (%)
Patients (number)	362
Age (year)	64.57 ± 11.79
Sex (Male)	233 (64.36)
Urban environment	190 (52.6)
**Type of Acute Coronary Syndrome**	
ST-elevation myocardial infarction	167 (46.13)
Non-ST elevation myocardial infarction	64 (17.68)
Unstable angina	131 (36.2)
**Cardiovascular Risk Factors and Comorbidities**	
Smoking	122 (33,7)
Previous MI	78 (21.55)
Hypertension	254 (70.16)
Grade I	30 (8.29)
Grade II	196 (54.14)
Grade III	28 (7.73)
Dyslipidemia	274 (75.7)
Chronic heart failure (according to New York Heart Association functional classification of heart failure, NYHA)	184 (50.82)
I	12 (3.31)
II	118 (32.59)
III	44 (12.15)
IV	12 (3.31)
Valvular heart disease	229 (63.26)
Chronic kidney disease	103 (28.45)
Diabetes type 2	141 (38.95)
Oral therapy	69 (19.06)
Insulin	72 (19.89)
History of stroke	27 (7.45)
**ECG Territory Changes**	
Anterior	98 (27.07)
Anterior and inferior	14 (3.87)
Anterior and lateral	10 (2.76)
Anterior septum	16 (4.41)
Inferior	64 (17.68)
Inferior and right leads	8 (2.21)
Inferior and lateral	16 (4.42)
Posterior and inferior	4 (1.10)
**Echocardiography**	
Left ventricular ejection fraction	40.45 ± 6.73
**Coronary Angiography**	
Not performed	159 (43.92)
Single-vessel disease	83 (22.92)
Left anterior descending artery	54 (14.9)
Circumflex artery	6 (1.6)
Right coronary artery	23 (8.8)
Two-vessel disease	70 (19.34)
Left main	6 (1.7)
Left anterior descending artery—circumflex coronary artery	30 (8.3)
Left anterior descending artery—right coronary artery	20 (5.5)
Circumflex coronary artery—right coronary artery	14 (3.9)
Three-vessel disease	50 (13.8)
**Myocardial Revascularization Procedure**	
Thrombolysis	38 (10.5)
Interventional or surgical revascularization	
Percutaneous coronary intervention	176 (48.62)
Coronary artery bypass graft	4 (1.10)
Not performed	182 (50.28)

**Table 2:** In-hospital complications of patients with acute coronary syndrome, at their first admission.

In-Hospital Complications	No (%)
**Rhythm disturbances**	95 (26.24)
Atrio-ventricular block, first degree	2 (0.55)
Atrio-ventricular block, first degree + left bundle branch block	2 (0.55)
Third-degree atrio-ventricular block	2 (0.55)
Left bundle branch block	4 (1.1)
Premature atrial beats	12 (3.31)
Premature ventricular beats	13 (3.59)
Paroxysmal atrial fibrillation	4 (1.1)
Persistent atrial fibrillation	38 (10.5)
Atrial fibrillation + third-degree atrio-ventricular block	2 (0.55)
Ventricular tachycardia non-sustained	4 (1.1)
Sustained ventricular tachycardia	6 (1.66)
Ventricular fibrillation	6 (1.7)
Acute pulmonary edema	15 (4.14)
Cardiogenic shock	20 (5.52)
Resuscitated cardiac arrest	15 (4.14)
Upper gastrointestinal bleeding	6 (1.65)
**Acute stroke**	6 (1.65)
Ischemic	2 (0.55)
Hemorrhagic	4 (1.1)

**Table 3:** Comparison between patients with early readmission (in the next 12 months following the initial event) and late readmission (>12 months after the initial event).

Demographic Parameters	Readmission < 12 Months—No. (%)	Readmission > 12 Months—No. (%)	*p*
	201 (55.52)	161 (44.48)	
Age (Y)	64.23 ± 10.871	64.18 ± 12.363	0.969
Sex (F)	76/201 (37.81)	53/161 (32.92)	0.432
**Type of Acute Coronary Syndromes at First Admission**			
ST-elevation myocardial infarction	93/201 (46.28)	74/161 (45.96)	0.954
non-ST elevation myocardial infarction	39/201 (19.40)	25/161 (15.53)	0.415
Unstable angina	70/201 (34.83)	61/161 (37.89)	0.485
**Risk Factors and Comorbidities**			
History of previous myocardial infarction	41/201 (20.39)	37/161 (22.98)	0.514
Hypertension	141/201 (70.14)	113/161 (70.19)	0.62
Dyslipidemia	154/201 (76.61)	120/161 (74.5)	0.497
Diabetes	81/201 (40.29)	60/161 (37.26)	0.682
Chronic kidney disease	65/201 (32.33)	38/161 (23.60)	0.055
Chronic heart failure	115/201 (57.21)	69/161 (42.86)	0.191
Acute pulmonary edema at first admission	13/201 (6.47)	2/161 (1.24)	0.022 *
Cardiogenic shock at first admission	15/201 (7.46)	5/161 (3.11)	0.056
Valvular heart disease	139/201 (69.15)	90/161 (55.90)	0.017 *
Left ventricular ejection fraction %	39.20 ± 6.85	42.24 ± 6.26	<0.001 *
Arrhythmias	53/201 (26.37)	42/161 (26.09)	0.889
History of stroke	15/201 (7.46)	12/161 (7.45)	0.541
Upper gastrointestinal bleeding	4/201 (1.99)	2/161 (1.24)	0.594
Resuscitated cardiac arrest	11/201 (5.47)	4/161 (2.48)	0.052
Thrombolysis at first admission	18/201 (8.96)	20/161 (12.42)	0.262
**Coronarography Characteristics at First Admission**			
Single-vessel disease	39/201 (19.40)	44/161 (27.33)	0.065
Two-vessel disease	40/201 (19.9)	30/161 (18.63)	0.806
Three-vessel disease	38/201 (18.90)	12/161 (7.45)	0.002 *
**Revascularization Type of Procedure at First Admission**			
Percutaneous coronary intervention	102/201 (50.74)	74/161 (45.96)	0.81
Coronary artery bypass graft	-	4/161 (2.48)	-
Complete revascularization at first admission	50/201 (24.87)	56/161 (34.78)	0.005 *

* *p* values < 0.05.

**Table 4:** Significant correlations between parameters (at index hospitalization) and acute coronary syndrome type (at the second admission).

Significant Correlations for ST-Elevation Myocardial Infarction	*p*	r
Three-vessel disease	0.034	0.364
Left ventricular ejection fraction	0.013	0.359
Upper gastrointestinal bleeding at first hospitalization	0.012	0.361
Unstable angina at first hospitalization	0.002	0.361
**Significant Correlation for Non-ST Elevation Myocardial Infarction**		
Culprit-lesion-only revascularization	0.028	0.351
Two-vessel coronary artery disease	0.018	0.361
Previous myocardial infarction	0.004	0.361
Upper gastrointestinal bleeding at first hospitalization	0.005	0.361
**Significant Correlation for Unstable Angina Pectoris**		
Heart failure	0.005	0.361
Left ventricular ejection fraction	0.002	0.359
Chronic kidney disease	0.0018	0.361

## Data Availability

Data of the patients are available in the archive of the hospital in the study.
